# Peripheral intravenous cannulation with support of infrared laser vein viewing system in a pre-operation setting in pediatric patients

**DOI:** 10.1186/s13104-015-1431-2

**Published:** 2015-09-21

**Authors:** Andreas Rothbart, Peng Yu, Lutz Müller-Lobeck, Claudia D. Spies, Klaus-Dieter Wernecke, Irit Nachtigall

**Affiliations:** Department of Anesthesiology and Intensive Care Medicine Campus Charité Mitte and Campus Virchow-Klinikum, Charité-Universitaetsmedizin Berlin, Augustenburger Platz 1, 13353 Berlin, Germany; Department of Anesthesiology, The Second Affiliated Hospital of Jiaxing University, Jiaxing, China; Department of Anesthesiology and Intensive Care Medicine, Pediatric Anesthesia, Clinical Center Barnim, Werner Forssmann Hospital, Eberswalde, Germany; Institute of Medical Biometrics and Clinical Epidemiology, Charité-Universitaetsmedizin Berlin, and SOSTANA GmbH Berlin, Berlin, Germany

**Keywords:** Cannulation, Venipuncture, Induction, Anesthesia-pediatric, EMLA^®^

## Abstract

**Background:**

Venous access, a prerequisite for anesthesiological and surgical intervention in pediatric patients, is often difficult to establish and potentially painful. AV300 uses near infrared laser light to improve visibility of peripheral veins and could help cannulating them. The aim of this study was to examine if use of Accuvein^®^ AV300 vein viewer could facilitate venous cannulation in children.

**Methods:**

From January to March 2011, 238 consecutive pediatric patients (0–17 years) preceding surgical interventions were included. All participants including newborns, infants and children were allocated to groups [control group (124 patients) and intervention group (114 patients)] in a non-random way. Randomization was not feasible because data was acquired retrospectively from a clinical quality management project. In control group, peripheral IV cannulation was performed without supporting device, in intervention group with support of AV300. Time and number of attempts until successful venous cannulation were defined as primary end points.

**Results:**

Median time until successful cannulation was 2 min (range 0.1–20, quartiles: 25 %: 1; 75 %: 5) in the intervention group and 1 min (range 0.1–18, quartiles: 25 %: 0.2; 75 %: 2) in the control group (p < 0.01). Median number of attempts was higher in the intervention group (2; range 1–6, quartiles: 25 %: 1; 75 %: 3) than in the control group (1; range 1–6, quartiles: 25 %: 1; 75 %: 2, p < 0.01). Rate of cannulations successful at first attempt was 0.45 (51 of 114, 95 % CI 0.35–0.54) in the intervention group and 0.73 (90 of 124, 95 % CI 0.65–0.81) in the control group (p < 0.01).

**Conclusions:**

In our study we were not able to reduce neither time nor number of attempts until a successful venous cannulation in children using the vein viewer. Given certain limitations of our study as the lack of randomization and no control for inter-operator variability, the conclusions drawn from it are also limited, but by our results laser-supported cannulation cannot be recommended for standard procedures.

Trial registration: ClinicalTrials.gov NCT01434537. Registered 29 July 2011

## Background

In many clinical situations a fast and effective venous access is essential for patient’s safety and care: for rehydration, in emergency situations, application of systemic drugs and for every anesthesiological procedure. While inhalational anesthesia induction in children has been standard care throughout the world for many years, the prerequisite of a peripheral venous access prior to the induction becomes more and more part of clinical practice, at least in elective procedures. The aim is to establish the venous access fast, with few attempts and without causing pain in the pediatric patients. It has been shown that there are effective ways to reduce pain [1] by applying local anesthetics on the puncture site prior to puncture.

In many pediatric patients the venous access is difficult to establish due to their thicker layer of subcutaneous tissue compared to adults, particularly in children younger than 3 years [2]. In these cases the prolonged procedure can result in more pain, trauma and subcutaneous hemorrhage from frequent attempts. For the latter, people tried to use different methods to dilate the subcutaneous vein to enhance visibility, and facilitate cannulation [3–5], and—more important—many viewing system were invented to facilitate the locating of superficial veins at a peripheral site [6–9]. The AccuVein^®^ AV300 acts as one of the vein imaging techniques using near-infrared (NIR) light [9–13]. It is a portable, non-contact vein viewer for improving the detection of subcutaneous veins. NIR light emitted from the imaging system is used to locate the vein to be punctured. However, up to now and to our knowledge, few reports are available about its effects on subcutaneous vein cannulation in pediatric patients in a pre-operation setting. This trial aims to investigate whether the AV300 could reduce time until and number of attempts for successful peripheral vein cannulation in pediatric patients in a pre-operation setting.

## Methods

The study was approved by the IRB of Charité (EA1/171/11) and registered at clinicalTrials.gov (ID: NCT01434537) on July 29th, 2011. The ethics committee waived the need for written informed consent for the intervention group, since this retrospective study was performed as an analysis of a project accompanying the introduction of an already approved device into clinical standard of our department. The project was initiated from the Department of Anesthesiology and Intensive Care CVK/CCM, Charité-Universitaetsmedizin Berlin, and not from the manufacturer of the device. No data were handed out to the manufacturer. Only because the project revealed information that seemed to be important to publish, the ethics committee was asked whether to retrospectively analyze and publish the anonymous data and agreed to do so. From January 2011 altogether 238 consecutive pediatric patients from 0 to 17 years of age preceding surgical interventions in the pediatric operating room of the Campus Virchow-Klinikum, Charité-Universitaetsmedizin Berlin, were included in the trial. All of the participants including newborns, infants and children were allocated to two groups in two timeframes of each 4 weeks: The first consecutive 114 patients were allocated to the intervention group, the next 124 into the control group. Randomization was not feasible because data was acquired retrospectively from a clinical quality management project. In the control group, peripheral IV cannulation was performed as usual without any supporting device. In the intervention group, the same intervention as in the control group was done but with support of AV300 vein viewer (AccuVein^®^, LLC, 40 Goose hill Rd, Cold Spring Harbour, NY) following the User Manual of AccuVein^®^ model AV300 vein viewing system [13]. During IV insertion, the AV300 was held by an assistant perpendicularly about 18 cm over the patient’s limb where the vein was expected to be located. Sometimes, in order to get a good visualization of the target vein, the height or the angle of the device had to be slightly altered. The AV300 should not beam directly in the eyes because the device emits two Class 2 lasers, a red one with the wavelength of 642 nm and a near-infrared one at 785 nm. Due to the absorption of the near-infrared light by hemoglobin, the vein can be visualized [13]. Before the start of this study doctors and nurses were trained for the use of the vein viewer by an anesthetist that was trained by the manufacturer. It is common practice in our clinic that everyone must be trained for every device before using it the first time on patients.

The catheters used for venous cannulation were BD Insyte Autoguard Winged 24GA from Becton–Dickinson Infusion Therapy Systems Inc., Sandy, Utah, USA; BD Neoflon 24GA and 26GA, BD Venflon Pro Safety 22GA from Becton–Dickinson Infusion Therapy AB, Helsingborg, Sweden.

All IV cannulations were performed by skilled and experienced anesthesiologists, residents and nurses. The time from the beginning of the first cannulation attempt (first skin-needle contact) was measured by a member of the research team. The end of the procedure was defined as the successful establishment of a vein access easily flushable by sterile 0.9 % sodium chloride solution.

Data were collected on gender, age, weight, presence of general anesthesia or not, received local anesthesia (EMLA^®^, a mixture of lidocaine and prilocaine) or not, needle size, the time and number of attempts until successful cannulation for both groups.

Data were analyzed by SPSS for windows version 18 (SPSS Inc., Chicago, IL). Results were given by rates (in %) with 95 % confidence limits as well as by median and 25–75 %-quartiles for continuous variables because of deviations from normal distribution. Chi square and nonparametric (Mann–Whitney) testing was used to access the differences in demographic data (gender, age, weight, received general anesthesia or not, received local anesthesia or not, needle size, first-attempt success rate) between both groups. Explorative data analysis including checks for normal distribution (P–P plots) was applied. Time and number of attempts until successful venous cannulation were defined as primary end points. Differences in outcome variables were analyzed by nonparametric tests (Mann–Whitney-U-test). Rates were tested using the Chi square test. P-values <0.05 were considered statistically significant. All tests should be understood as constituting exploratory data analysis, such that no adjustments for multiple testing have been made.

## Results

Basic characteristics: There were 238 pediatric patients involved in the trial, 124 in the control group, and 114 in the intervention group. The youngest patient was <1 month; the oldest 17 years and 5 months old, mean age was 48.4 (median: 24) months. Differences between both groups were tested with Chi square-test as well as Mann–Whitney-tests and there were no statistically significant differences found in distribution of gender, age, weight and presence of general anesthesia. There were no differences found in the sizes of the used needles (p = 0.069). The only difference in this main group was that in the intervention group the proportion of patients with applied local anesthetics (EMLA^®^) was higher (p = 0.003) (Table 1).

**Table 1:** Basic characteristics

	Control group count (% within groups) or median (IQR), n = 124	Intervention group count (% within groups) or median (IQR), n = 114	
Gender			
Male	72 (58.1)	59 (51.8)	p = 0.362
Female	52 (41.9)	55 (48.2)	
Age (months)			
0–3	12 (9.7)	19 (16.7)	p = 0.346
4–12	24 (19.4)	28 (24.6)	
13–24	22 (17.7)	15 (13.2)	
25–72	28 (22.6)	23 (20.2)	
≥73	38 (30.6)	29 (25.4)	
Weight (kg)			
<5	9 (7.3)	11 (9.6)	p = 0.538
5–9.99	37 (29.8)	41 (36.0)	
10–19.99	38 (30.6)	29 (25.4)	
20–39.99	24 (19.4)	24 (21.1)	
≥40	16 (12.9)	9 (7.9)	
General anaesthetic (sevoflurane or N_2_O)			
No	52 (41.9)	41 (36.0)	p = 0.356
Yes	72 (58.1)	73 (64.0)	
Local anaesthetic (EMLA^®^)			
No	67 (54.0)	39 (34.2)	p = 0.003
Yes	57 (46.0)	75 (65.8)	
LA in >72 months			
No	28 (73.7)	6 (20.7)	p < 0.001
Yes	10 (26.3)	23 (79.3)	
LA in ≤72 months			
No	39 (45.3)	33 (38.8)	p = 0.440
Yes	47 (54.7)	52 (61.2)	
Size of needles (GA)			
26	0 (0)	3 (2.6)	p = 0.069
24	59 (47.6)	63 (55.3)	
22	57 (46.0)	38 (33.3)	
20	8 (6.5)	10 (8.8)	

*IQR* interquartile range, *GA* Gauge, *LA* local anesthetic, *EMLA* ^*®*^ Eutectic Mixture of Local Anesthetics

**Fig. 1:**
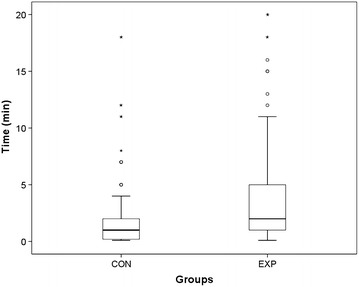
Time until successful venous cannulation (*CON* control; *EXP* experimental group)

Median time until successful venous cannulation was 2 min (range 0.1–20, quartiles: 25 %: 1; 75 %: 5) in the intervention group and 1 min (range 0.1–18, quartiles: 25 %: 0.2; 75 %: 2) in the control group (p < 0.01) (Fig. 1).

**Fig. 2:**
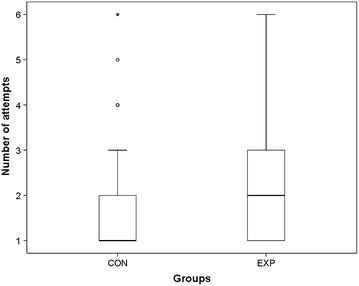
Number of attempts until succesful venous cannulation (*CON* control; *EXP* experimental group)

Median number of attempts was higher in the intervention group (2; range 1–6, quartiles: 25 %: 1; 75 %: 3) than in the control group (1; range 1–6, quartiles: 25 %: 1; 75 %: 2, p < 0.01) (Fig. 2). The rate of cannulation which were successful after the first attempt was 0.45 (51 of 114, 95 % CI 0.35–0.54) in the intervention group and 0.73 (90 of 124, 95 % CI 0.65–0.81) in the control group (p < 0.01).

**Fig. 3:**
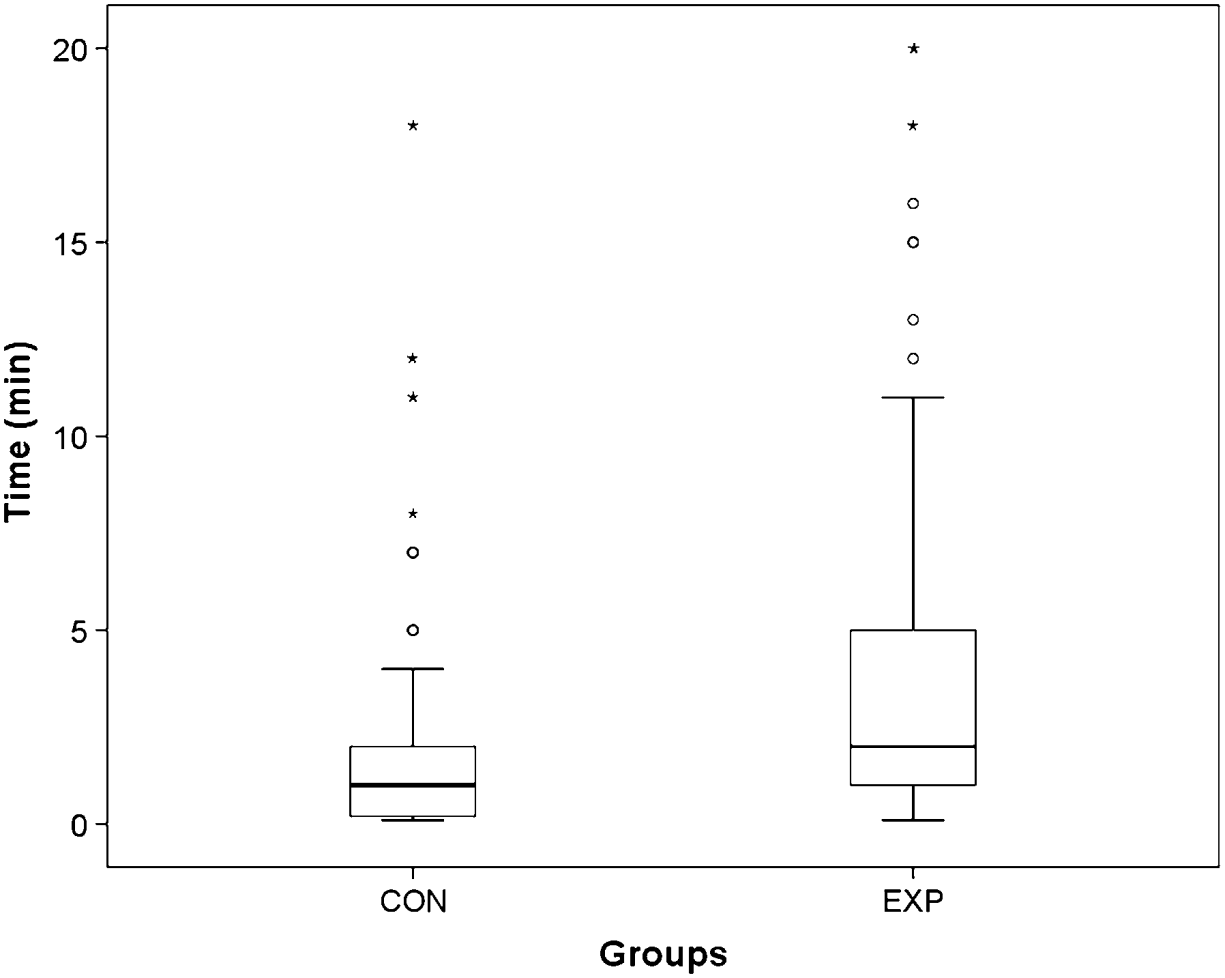
Time until successful venous cannulation in subgroup up to 72 months (*CON* control; *EXP* experimental group)

**Fig. 4:**
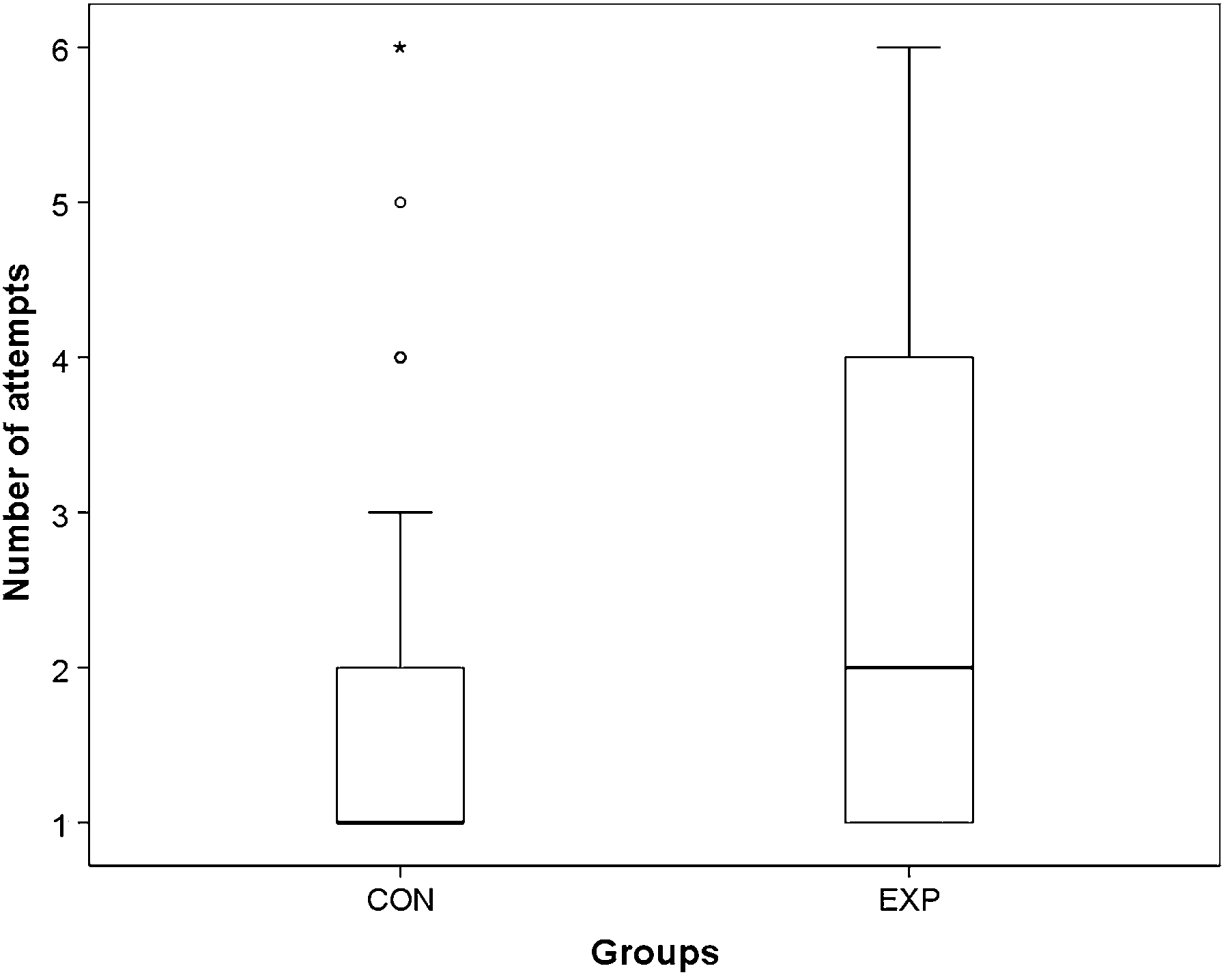
Number of attempts until succesful venous cannulation in subgroup up to 72 months (*CON* control; *EXP* experimental group)

If the subgroup of 171 patients of 72 months and younger is regarded, there was no difference in the use of EMLA^®^ between the two groups. In this subgroup median time until cannulation was 1 min (range 0.1–18, quartiles: 25 %: 1; 75 %: 2) in the control and 2 min (0.1–20, quartiles: 25 %:1; 75 %: 6) in the experimental group (p < 0.01) (Fig. 3). The median number of attempts was higher in the intervention group (2, range 1–6, quartiles: 25 %: 1; 75 %: 4) than in the control group (1, range 1–6, quartiles: 25 %: 1; 75 %: 2; p < 0.01) (Fig. 4). The rate of cannulation which were successful after the first attempt was 0.38 (32 of 85, 95 % CI 0.27–0.48) in the intervention group and 0.66 (57 of 86, 95 % CI 0.56–0.76) in the control group (p < 0.01).

## Discussion

The results of this trial indicate that in our study group use of the Accuvein^®^ AV300 vein scanner was not able to reduce neither the time needed for establishing a peripheral venous access nor the needed number of puncturing attempts.

Our findings are comparable to those of a trial examining a similar device in an emergency room setting where the use of the vein viewer could not facilitate the venipuncture either [14]. Though another study group found that in a similar setting the use of such a device may decrease time of venous cannulation in children age 0–2 years [15].

These results differ substantially from a recent study showing that a vein viewing system similar to the device used in our study was able to facilitate venous cannulation of peripheral veins in difficult veins [16]. A study with a randomized-controlled design on another non-contact vein viewing system using the technique of transillumination reported a good visibility of peripheral veins, higher success rates at the initial attempt and less time taken until successful peripheral venous cannulation [7].

The use of local anesthetics like EMLA^®^ might have an influence on the results of these studies as it was reported that EMLA^®^ could cause vasoconstriction of the peripheral veins and pallor or edema at the application site and therefore make vein cannulation more difficult [17, 18]. Though one investigation indicated the skin-related side effects of EMLA^®^ were mild and transient, and might not have an influence on vein cannulation [19], EMLA^®^ is still thought to be one of the potential confounders in our trial. In our study our results seemed to point in the same direction, with EMLA^®^ being in a higher proportion in the intervention group, but when analyzing a subgroup of children of 72 months and younger there was no significant difference in the use of EMLA^®^ (Table 1). Neither in this group without the EMLA^®^ difference, the use of the AccuVein^®^ AV300 was able to reduce neither the time nor the number of attempts until successful cannulation of peripheral veins either.

Another product designed on the basis of NIR (VeinViewer^®^, Luminetx Corporation, Memphis, Tenn.), presented prominent value in facilitating venipuncture and IV catheterization [9, 20, 21]. A clinical observation reported a high rate of satisfaction from fifty questionnaires was but there was no control group [20]. Another investigator argued in a recent publication that no benefit was shown for the first-attempt success rate during the pediatric IV cannulation with aid of VeinViewer^®^ in a randomized controlled trial [14]. Recently Szmuk et al. reported that the use of the same device could even worsen the first-attempt cannulation rate in a randomized trial [22].

In our study, errors in size and position between the vein shadow and the vein itself may have contributed to the outcome. In some cases, it was difficult to get an IV insertion due to the enlarged vein images with aid of the AV300; meanwhile, a failing detection of the target vein depth was thought to be an obvious disadvantage. In addition, the target site chosen for IV insertion was just exposed in the NIR beam and punctured directly, often leading to deformation of the vessel image by the needle pressing. So the endeavor to avoid the deformation of the vein image would offer one of the solutions to improve the first-attempt success rate as using the NIR devices [20]. A recent study found that a similar device could facilitate the venous access in difficult cases [16]. A recently published cluster randomized trial came to the conclusion that even if vein visibility was enhanced, near-infrared devices do not improve cannulation [23].

The main limitation of our trial is the lack of randomization which causes a bias. The sample size was large compared to similar studies but might have still been too small. The influence of the person practicing the venipuncture was limited by the fact that only experienced persons were in charge but this fact could be controlled better by stratifying the staff or switching after the half of the study into the other group. Observer bias could have been caused by the fact that the member of the research team measuring the time until successful cannulation was not blinded. A dummy vein viewer would need to have been used in the control group to control for. The comorbidities and ASA classification of the patients should also be taken into account in a future study to clarify the benefit and utility of these devices based on NIR.

## Conclusions

In our study the use of the Accuvein^®^ Vein Viewer was not able to reduce neither time nor number of attempts until a successful venous cannulation. Thus, its use in standard procedures with easy cannulations cannot be recommended within the limitations of this study.

Even if the findings of these studies including the present examining the benefit of vein viewing systems are inconsistent, there might be a place for using them in cases where peripheral veins are poorly visible or inexperienced medical staff performs the cannulation.

## Abbreviations

ASA: American Society of Anesthesiologists
CCM: Campus Charité Mitte
CI: confidence interval
CVK: Campus Virchow-Klinikum
EMLA^®^: Eutectic Mixture of Local Anesthetics)
GA: Gauge
IQR: interquartile range
IRB: institutional review board
IV: intravenous
LA: local anesthetic
NIR: near-infrared
